# Cry of Foetus in Utero

**Published:** 1913-09

**Authors:** 


Cry of Foetus in Utero. W. P. Moncure of Fairfax, Va., *Homeopathic Recorder,* April, 1913, reports a case.

